# Inhibition of mTOR-kinase destabilizes MYCN and is a potential therapy for MYCN-dependent tumors

**DOI:** 10.18632/oncotarget.10544

**Published:** 2016-07-12

**Authors:** Lynsey Vaughan, Paul A. Clarke, Karen Barker, Yvan Chanthery, Clay W. Gustafson, Elizabeth Tucker, Jane Renshaw, Florence Raynaud, Xiaodun Li, Rosemary Burke, Yann Jamin, Simon P. Robinson, Andrew Pearson, Michel Maira, William A. Weiss, Paul Workman, Louis Chesler

**Affiliations:** ^1^ Division of Clinical Studies, The Institute of Cancer Research, Sutton, Surrey, UK; ^2^ Cancer Research UK Cancer Therapeutics Unit, The Institute of Cancer Research, Signal Transduction and Molecular Pharmacology Team, The Institute of Cancer Research, Sutton, Surrey, UK; ^3^ Department of Neurology, Pediatrics, Neurosurgery, Brain Tumor Research Center and Helen Diller Family Comprehensive Cancer Center, University of California, San Francisco, CA, USA; ^4^ Cancer Research UK Cancer Therapeutics Unit, Clinical Pharmacology and Trials Team, Sutton, Surrey, UK; ^5^ Cancer Research UK Cancer Therapeutics Unit, The Institute of Cancer Research, Target Selection and Hit Discovery Team, The Institute of Cancer Research, Sutton, Surrey, UK; ^6^ Cancer Research UK & Engineering and Physical Sciences Research Council Cancer Imaging Centre, The Institute of Cancer Research, Sutton, Surrey, UK; ^7^ Novartis Pharma AG, Basel, Switzerland; ^8^ The Royal Marsden NHS Trust, Children and Young People's Unit, Sutton, Surrey, UK; ^9^ Present address: Cell Signalling Group, Cancer Research UK Manchester Institute, The University of Manchester, Manchester, UK; ^10^ Present address: Basilea Pharmaceutica International AG, Basel, Switzerland; ^11^ Present address: MRC Cancer Unit, University of Cambridge, Cambridge, UK

**Keywords:** neuroblastoma, mTOR, MYC, MYCN, PI3-kinase

## Abstract

MYC oncoproteins deliver a potent oncogenic stimulus in several human cancers, making them major targets for drug development, but efforts to deliver clinically practical therapeutics have not yet been realized. In childhood cancer, aberrant expression of *MYC* and *MYCN* genes delineates a group of aggressive tumours responsible for a major proportion of pediatric cancer deaths. We designed a chemical-genetic screen that identifies compounds capable of enhancing proteasomal elimination of MYCN oncoprotein. We isolated several classes of compound that selectively kill MYCN expressing cells and we focus on inhibitors of PI3K/mTOR pathway in this study. We show that PI3K/mTOR inhibitors selectively killed MYCN-expressing neuroblastoma tumor cells, and induced significant apoptosis of transgenic MYCN-driven neuroblastoma tumors concomitant with elimination of MYCN protein *in vivo*. Mechanistically, the ability of these compounds to degrade MYCN requires complete blockade of mTOR but not PI3 kinase activity and we highlight NVP-BEZ235 as a PI3K/mTOR inhibitor with an ideal activity profile. These data establish that MYCN expression is a marker indicative of likely clinical sensitivity to mTOR inhibition, and provide a rationale for the selection of clinical candidate MYCN-destabilizers likely to be useful for the treatment of MYCN-driven cancers.

## INTRODUCTION

Aberrant expression of the transcription factors MYC and MYCN (a MYC homologue with expression limited to undifferentiated neurons) delivers a potent oncogenic stimulus in cancer, making MYC oncoproteins attractive targets for pharmacologic inhibition [1–7]. In pediatric cancer, expression of MYCN is selectively confined to tumor tissue in medulloblastoma [8], neuroblastoma [1, 2], rhabdomyosarcoma [7], and a subset of retinoblastoma [9], four common solid tumors that together account for a substantial proportion of relapsed cancer deaths in children. Pharmacologic inhibition of MYCN oncoprotein is therefore of great interest in pediatric cancer; however, direct targeting of MYCN, or MYC oncoproteins in general, has not yet delivered viable therapeutics to the clinic.

A substantial body of work suggests two approaches by which MYC proteins could be therapeutically targeted. First, synthetic lethal interactions are generated by persistent overexpression of MYC in cancer cells and impart therapeutic sensitivity [10–16]. Genetic screens utilizing RNA-interference have identified relationships between expression of MYC and a defined set of additional genes, suppression of which induces lethality restricted to cells with MYC or MYCN expression (*CDK1, CDK2, AURKA, AURKB, CHK1*) [10–12, 17–23]. Second, MYC-family oncoproteins are oncogenically stabilized by altered phosphorylation within an N-terminal conserved phosphodegron domain (CPD) [14, 24–26]. Binding to the CPD is a function of the ubiquitin ligases (FBW7, HUWE1) and is required for initiation of proteasomal degradation [4, 24, 27]. Phosphorylation of conserved T58 and S62 residues within the CPD is regulated by CDK1 (a MAPK target), and by the PI3K/AKT-regulated targets GSK3β and mTOR, respectively [28–30]. Cancers defined by mutations in FBW7 (which disrupt the association of FBW7 with the CPD-client protein) are characterized by elevated levels of the CPD-client oncoproteins MYC [31], c-Jun [32], cyclin E [33] and NOTCH [34]. Aberrant stimulation of MAPK or PI3K pathways also contributes to oncogenic stabilization of MYCN in certain cancers. In neuroblastoma, enhanced PI3K signaling correlates with poor prognosis and aggressive tumor biology [35, 36]. Previous work established that in cancer cell lines, excess PI3K pathway signaling modulates the AKT-regulated targets GSK3β and mTOR, extending the half-life of MYC and outlining a potential pharmacologic approach for targeting MYC stability [28–30, 34, 37].

To our knowledge, a chemical-genetic screen that addresses these two mechanisms, corroborating previous synthetic lethality studies of MYC oncogene expression or providing the targets for MYC oncoprotein destabilization, has not been reported. With this in mind, we designed a cell-based screen utilizing an isogenically derived set of neuroblastoma cells with either high-level expression of wild-type MYCN or with genetically modified, stabilized MYCN (lacking the CPD target phosphorylation residues, T58 and S62). Potent compounds that have mechanistic activity in cells expressing wild-type, but not CPD mutated, MYCN protein should antagonize oncogenic stabilization of MYCN protein and target synthetic lethal relationships associated with MYCN expression. We outline a strategy to identify compounds that specifically target wild-type MYCN protein and present a chemical screen that identifies small-molecules with the ability to indirectly pharmacologically target MYCN (and potentially MYC) protein. Compounds inhibiting several genetic pathways were identified in the screen and in this study we characterize small-molecule inhibitors of the PI3K/mTOR pathway. We show that inhibitors of PI3K/mTOR, or mTOR kinase activity alone, exhibit selective activity against cells that express high-levels of MYCN protein. NVP-BEZ235, a potent inhibitor of both PI3K and mTOR-kinases, which is already in clinical trials for PI3K pathway-mutated adult cancers, eliminated intra-tumoral MYCN and induced regression of MYCN-driven transgenic neuroblastoma tumors *in vivo*.

Overall our observations raise the exciting possibility that a group of poor-outcome pediatric cancers could be effectively targeted through their expression of a tumor-specific oncoprotein, using available and clinically tested therapeutics.

## RESULTS

### Screen for small-molecules that target MYCN oncoprotein stability

We designed a focused chemical-genetic screen that would identify small-molecules with a mechanistic ability to target cells expressing high-levels of wild-type MYCN. Expression constructs encoding FLAG-tagged wild type or stabilized (CPD-mutated) MYCN proteins were transfected into human neuroblastoma cells lacking endogenous MYCN expression. Equivalent levels of MYCN expression were achieved in SHEP cells expressing wild-type MYCN (SHEP WT) in comparison to a range of established, tumor-derived neuroblastoma cell lines known to express high-levels of MYCN (Figure S1A). However, elevated levels of MYCN expression were observed in SHEP cells expressing mutated MYCN (SHEP T58, SHEP S62 and SHEP T58/S62) in comparison with both SHEP WT and established neuroblastoma cell lines - consistent with the stabilization of CPD-mutated MYCN (Figure S1A, B). SHEP cells expressing both wild type and mutant MYCN proteins all exhibited significantly increased cellular proliferation in comparison to parental SHEP cells but not to each other (Figure S1C).

To establish the MYCN dependency of both the native neuroblastoma cell line Kelly and the SHEP WT and SHEP T58/S62 mutant MYCN lines, we abrogated MYCN expression *via* siRNA-mediated knockdown. Inhibition of MYCN in the Kelly cell line induced cell death as measured by trypan blue exclusion (Figure S1D) and similarly, although to a lesser extent, MYCN cell viability was diminished with siRNA treatment in both SHEP WT and SHEP T58/S62 neuroblastoma cell lines (Figure S1E, F), confirming that exogenous MYCN expression is responsible for the increased proliferation observed in SHEP WT and SHEP T58/S62 cells.

**Figure 1:**
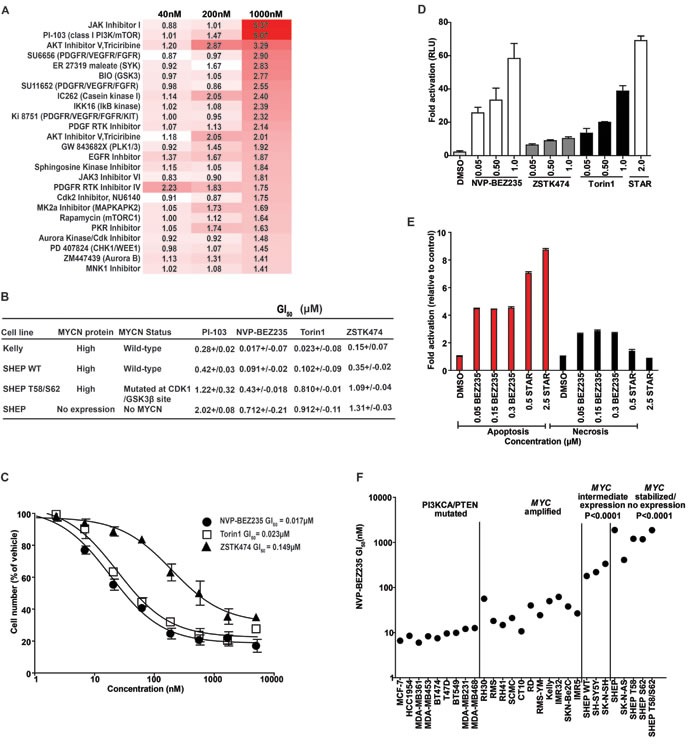
Identification of PI3K/mTOR inhibitors that selectively target MYCN-expressing tumor cells **A.** SHEP WT and SHEP T58/S62 cells were treated at a concentration of 40, 200 and 1000nM for 96 h with a panel of 228 kinase inhibitors exhibiting a range of kinome inhibitory properties. Cell viability was determined using CellTiter-blue reagent. The Z factor for all assay plates was >0.5. The data are displayed as a ratio of SHEP T58/S62:SHEP WT, increased red indicates increased activity in SHEP WT compared to SHEP T58/S62 cells. **B.** Cell viability as determined by trypan blue exclusion method in Kelly, SHEP, SHEP WT and SHEP T58/S62 neuroblastoma cells. Cells were treated for 72 h with PI-103, NVP-BEZ235, Torin1 or ZSTK474. Mean GI_50_ and standard error from three independent assays are shown. **C.** Representative log curves of Kelly cells treated for 72 h with, NVP-BEZ235, Torin1 or ZSTK474. Values represent the averages of three independent assays. Error bars; standard deviation. **D.** Induction of apoptosis 24 h post treatment with DMSO, NVP-BEZ235, ZSTK474, Torin1 or Staurosporine (as a positive control) in Kelly neuroblastoma cells as measured by Caspase-Glo 3/7 cleavage assay. Values are fold activation of caspase activity normalised to DMSO control and are averages of three assays. Error bars; standard deviation. **E.** Induction of apoptosis and necrosis by NVP-BEZ235. Kelly cells were treated with NVP-BEZ235 or Staurosporine (STAR) as a positive inducer of apoptosis and cell apoptosis and necrosis assessed via Cell Death ELISA (Roche™) 24 h post treatment. (Apoptosis; red bars and necrosis; black bars). Values are fold induction of histone-associated DNA fragments normalized to DMSO control and are averages of three assays. Error bars; standard deviation. **F.** Growth inhibitory (GI_50_s) values carried out at 72 h using the SRB assay of a panel of adult cancer cell lines carrying *PIK3CA/PTEN* mutations compared with pediatric cancer cell lines containing a spectrum of *MYCC/N* gene copy number or mutated *MYCN*. See also Figure 1B and Table S2.

Using cellular proliferation as an endpoint, we selected for compounds with enhanced activity against SHEP WT cells compared to SHEP T58/S62 cells expressing stabilized MYCN. We reasoned that this selection would enrich for compounds with mechanistic activity against MYCN but exclude compounds with generic activity related to inhibition of cell proliferation rather than MYCN stability. The screen was performed using an in-house kinase inhibitor library of 228 compounds at low, intermediate and high concentrations (40nM, 200nM and 1μM) to identify compounds that exhibit on-target effects whilst excluding the possibility of off-target effects exerted by kinase inhibitors at excessive concentrations (>1μM). The top 25 ranked inhibitors that showed selective inhibition of SHEP WT cells included inhibitors of JAK/STAT pathway, receptor tyrosine kinases (PDGFR), PI3K pathway (PI3K, AKT and mTOR), and cell cycle checkpoints (AURKA, AURKB, CDK, PLK, WEE1 and CHK1) (Figure 1A).

The specificity of 17 of the inhibitors in this list has been profiled against a panel of 300 protein kinases [38]. Several demonstrate off-target activity, with 6 (JAK Inhibitor I, SU6656, SU11652, JAK3 Inhibitor VI, PKR Inhibitor and Aurora Kinase/Cdk Inhibitor) inhibiting >25 kinases by >75%, and a further 3 (PDGF RTK Inhibitor, PDGF Receptor Tyrosine Kinase Inhibitor IV and Cdk2 Inhibitor IV-NU6140) inhibiting >5 kinases. Of these, 5 had activity against both Aurora A and B (Jak inhibitor I, SU6656, PDGF RTK inhibitor, PKR inhibitor, Aurora kinase/CDK inhibitor), Aurora A (Cdk2 Inhibitor IV-NU6140) or Aurora B (Jak3 VI inhibitor). The JAK inhibitor I also inhibited CHK1 and PLK1, CHK1 was also inhibited by SU11652 and the JAK3 VI inhibitor, and CDK1 was inhibited by the JAK3 VI inhibitor, PKR inhibitor and the Aurora Kinase/Cdk Inhibitor. The off-target and on-target activity of several of these inhibitors included AURA [12], AURB [39], CDK1 [10] and CHK1 [17] that have been reported to regulate MYCN stability or exhibit a synthetic lethal interaction with MYCN dependence. Our focused screen therefore appears to have strong predictive ability to identify compounds that are selective inhibitors of cells expressing MYCN protein. This chemically driven screen appears to validate previous RNAi driven loss-of-function studies, which highlighted the potential for pharmacologic targeting of synthetic lethal interactions in MYCN expressing cancers.

### PI3K pathway inhibitors selectively target cells with MYCN expression

Potent and selective inhibitors of PI3K and/or mTOR pathways have entered clinical trials in both adult and pediatric cancers, and could be valuable agents for treatment of childhood cancers [40, 41]. The fact that PI-103, a potent and highly specific small-molecule inhibitor of class I PI3K and mTOR [42–44] preferentially targeted MYCN-expressing cells in our screen was of particular interest to us. Preliminary studies demonstrated that LY294002, a first-generation, weak inhibitor of PI3K pathway that has off-target liabilities [45], destabilized MYCN and could partially inhibit the growth of MYCN-driven neuroblastoma tumors [46] but its use was limited by the off-target toxicity profile of LY294002, which restricted *in vivo* dosing. Given the activity of PI-103 (a more potent and selective inhibitor of PI3K signaling than LY294002) in our focused screen, and the availability of additional potent and selective PI3K inhibitors for clinical use, we focused on the role of PI3K/mTOR signaling in MYCN stability (Table S1).

We first re-confirmed our initial observation that the proliferation of SHEP WT cells was preferentially inhibited by PI-103 treatment using a trypan blue exclusion assay (Figure 1B). SHEP WT cells exhibited a 4.8-fold and 2.9-fold increased sensitivity to PI-103 compared to the parent SHEP cells or SHEP T58/S62 respectively. This differential sensitivity pattern was reproduced with NVP-BEZ235 [47], an imidazo-[4,5-c]-quinoline derivative PI3K and mTOR inhibitor (7.1 and 4.7-fold respectively), and also with Torin1 [48], an ATP-competitive mTOR-kinase (mTORC1 and mTORC2) inhibitor lacking PI3K inhibition, and to a lesser degree with ZSTK474 [49], a pan class I PI3K inhibitor that has poor activity against mTOR (3.8 and 3.2-fold respectively). In addition, the native neuroblastoma Kelly cells also exhibited a similar sensitivity profile as the SHEP WT cells (Figure 1B). These results show a clear trend in drug sensitivity where inhibition of cell proliferation aligns with the degree of *MYCN* amplification and protein expression. Our findings were reinforced both in an independent sulforhodamine B (SRB) assay of cell proliferation, and also in a larger cell panel that included four primary neuroblastoma cell lines with *MYCN* gene amplification, three cell lines with diploid *MYCN* and four engineered SHEP cell lines expressing mutated or wild-type exogenous MYCN protein (Table S2). Furthermore, TGX221, an isoform-selective p110β-selective PI3K inhibitor and rapamycin, a non-ATP site and incomplete inhibitor of mTOR (mTORC1-specific), showed relatively non-specific activity against this panel. The activity of NVP-BEZ235 and Torin1 against Kelly and three additional *MYCN*-amplified neuroblastoma cell lines correlated with induction of apoptosis in the Kelly cell line by two independent assays (Figure 1D, 1E). This data suggests that within the spectrum of available PI3K/mTOR pathway-active compounds, selective mTOR kinase inhibitors or dual inhibitors of PI3K and mTOR kinases are most likely to exhibit potent activity in neuroblastoma cells that express MYCN.

### Expression of MYCN selectively sensitizes cells to PI3K/mTOR inhibition

The enhanced sensitivity of MYCN overexpressing neuroblastoma cell lines to PI3K/mTOR inhibition is somewhat surprising, since these cells lack intrinsic mutations in the PI3K pathway members *PTEN* or PIK3CA. These mutations prime cells for response to PI3K pathway inhibitors and therefore provided the rationale for the initial use of these agents in clinical trials [50–52]. To examine whether MYCN expressing neuroblastoma cells exhibit similar sensitivity to cells harboring intrinsic PI3K pathway mutations we used NVP-BEZ235 to treat a range of adult cancer cell lines (*PTEN* or *PIK3CA* mutant, with varying expression levels of MYC [53–58]), together with pediatric cell lines (wild-type for PTEN and PIK3CA) but with varying expression levels of either MYC or MYCN (Figure 1F). We found that cell lines harboring no/intermediate expression of MYCN or with stabilized MYCN were significantly less sensitive to NVP-BEZ235 compared to both *MYC* and *MYCN*-amplified cell lines (*P* < 0.0001). Therefore, expression of MYCN is a marker of sensitivity to NVP-BEZ235 treatment in pediatric cell lines and confers sensitivity equivalent to that of adult cell lines mutated in *PTEN* or *PIK3CA*. Taken together, the implication is that expression of MYCN is a marker of sensitivity to PI3K/mTOR pathway inhibition in neuroblastoma cells.

**Figure 2:**
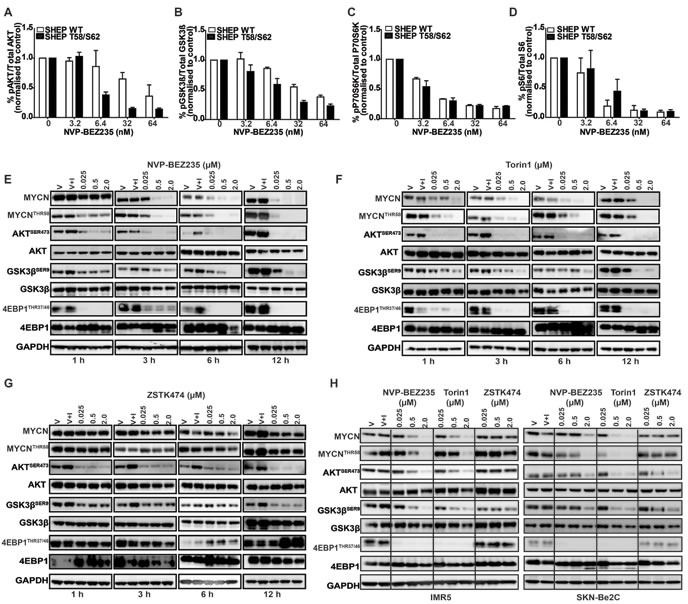
Inhibition of PI3K/mTOR signaling destabilizes MYCN **A.**-**D.** Mesoscale Discovery (MSD) analysis of PI3K/mTOR signaling components AKT^SER473^, GSK3β^SER9^, pP70S6K^THR389^ and RPS6^SER240/244^ SHEP WT and SHEP T58/S62 cell lysates were collected 3 h after treatment with NVP-BEZ235 and analysed for inhibitory effects on the phosphorylation of AKT^SER473^, GSK3β^SER9^, RPS6^SER240/244^ and pP70S6K^THR389^. Analysis was performed in triplicate using the MesoScale Discovery assay. Data are expressed as percent of control and values are shown as mean + SEM. **E.**-**G.** Immunoblotting analyses of time and concentration dependent effects of NVP-BEZ235, ZSTK474 and Torin1 treatment on MYCN stability and PI3K/mTOR biomarker AKT^SER473^, GSK3β^SER9^, 4EPB1^THR37/46^ modulation. Kelly cells were seeded and 24 h later treated with DMSO only (V), DMSO and IGF1 (V+I) or with NVP-BEZ235, Torin1 or ZSTK474 for the indicated time and concentrations. All inhibitor treated cells were stimulated with IGF1 (50ng/ml) 30 minutes prior to harvesting. AKT^SER473^, GSK3β^SER9^, 4EPB1^THR37/46^ MYCN^THR58^ and total AKT, GSK3β, 4EPB1 and MYCN were assessed *via* western blotting and GAPDH used as a loading control. **H.** Immunoblotting analyses of concentration dependent effects of NVP-BEZ235, ZSTK474 and Torin1 treatment on MYCN stability and PI3K/mTOR biomarker AKT^SER473^, GSK3β^SER9^ and 4EPB1^THR37/46^ modulation. IMR5 (Left panel) and SKN-Be2C (Right panel) neuroblastoma cells were seeded and 24 h later treated with DMSO only (V), DMSO and IGF1 (V+I) or with NVP-BEZ235, Torin1 or ZSTK474 at the indicated doses. All inhibitor treated cells were stimulated with IGF1 (50ng/ml) 30 minutes prior to harvesting. AKT^SER473^, GSK3β^SER9^, 4EPB1^THR37/46^ MYCN^THR58^ and total AKT, GSK3β, 4EPB1 and MYCN were assessed *via* western blotting and GAPDH used as a loading control.

Having identified selective sensitivity of MYCN-dependent cells to treatment with PI3K/mTOR inhibitors, we examined whether this could be explained by an intrinsic difference in PI3K pathway activation in these cells. We used an electro-chemiluminescent assay that quantitates PI3K/mTOR signaling intermediates (AKT^SER473^, GSK3β^SER9^, p70S6K^THR389^ and RPS6^SER240/244^) to compare PI3K/mTOR pathway inhibition in SHEP cells with exogenous expression of either MYCN WT or MYCN T58/S62 protein mutated within the CPD domain. NVP-BEZ235 caused potent blockade of PI3K and mTOR pathways in both SHEP WT and SHEP T58/S62 cell lines (Figure 2A-2D). Therefore the variance in viability between SHEP WT and SHEP T58/S62 cells following treatment with NVP-BEZ235 is unlikely to be due to differential inhibition of PI3K/mTOR pathway activity.

### Degradation of MYCN protein correlates with blockade of mTOR, not PI3K activity

**Figure 3:**
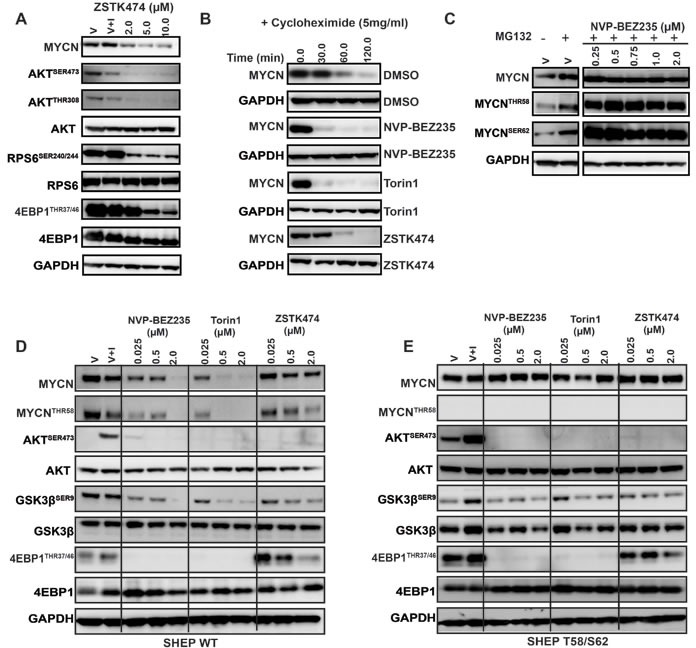
Inhibition of PI3K/mTOR signaling destabilizes MYCN **A.** Depletion of MYCN following treatment with high concentrations of ZSTK474, as measured by western blot analysis of AKT^SER473^, GSK3β^SER9^, 4EPB1^THR37/46^, RPS6^SER240/244^ and MYCN. Kelly cells were treated with DMSO (V), DMSO and IGF1 (V+I) or with ZSTK474, at the indicated concentration. All treated cells were stimulated with IGF1 (50ng/ml) 30 minutes prior to harvesting. Total AKT, RPS6 and 4EPB1 were used as controls for each corresponding phospho-specific antibody and GAPDH used as a loading control. **B.** Treatment of Kelly neuroblastoma cells with 1XGI_50_ concentration of NVP-BEZ235, Torin1 or ZSTK474 shortens the half-life of MYCN. Kelly neuroblastoma cells were treated with DMSO, NVP-BEZ235, Torin1 or ZSTK474 for 2 h before the addition of cycloheximide (5μM) for 1 h to block translation of MYCN. Total MYCN protein expression was assessed by western blotting, and GAPDH used as a loading control. **C.** Hyper-phosphorylation of T58/S62 phosphoresidues of MYCN is driven by PI3K/mTOR inhibition. Kelly neuroblastoma cells were treated with DMSO (V), DMSO and the proteasomal inhibitor MG-132, or with NVP-BEZ235 in the presence of MG132 for 3 h. Total, S62 and T58 pools of MYCN protein were assessed *via* western blot and GAPDH used as a loading control. **D.** Concentration dependent effects of NVP-BEZ235, Torin1 and ZSTK474 treatment on PI3K/mTOR pathway biomarkers AKT^SER473^, GSK3β^SER9^, 4EPB1^THR37/46^and MYCN stability in SHEP WT and (E) SHEP T58/S62 neuroblastoma cells. SHEP WT and SHEP T58/S62 cells were treated with DMSO (V), DMSO and IGF1 (V+I) or with NVP-BEZ235, Torin1 and ZSTK474 for 3 h. All treated cells were stimulated with IGF1 (50ng/ml) 30 minutes prior to harvesting. Total AKT, GSK3β, and 4EPB1 were used as controls for each corresponding phospho-specific antibody and GAPDH was used as a loading control.

We next sought to establish whether MYCN-selective inhibition observed in cellular assays correlates with elimination of MYCN protein, and if so, which component of PI3K pathway blockade is required for efficacy. To establish a time- and concentration-dependent biomarker response in a native neuroblastoma cell line we treated Kelly (*MYCN*-amplified, high MYCN protein) cells with NVP-BEZ235 for 1, 3, 6 and 12 h. Treatment led to a rapid (3 h) and sustained (12 h) elimination of MYCN protein by NVP-BEZ235 and Torin1 accompanied by a decrease in phosphorylation of 4EBP1^THR37/46^ (regulated by mTORC1) and AKT^SER473^ (regulated by mTORC2) (Figure 2E, 2F). Compared to NVP-BE235 and Torin1, there was no significant degradation of MYCN with ZSTK474 treatment, despite evidence of PI3K pathway inhibition indicated by a decrease in AKT^SER473^ and GSK3β^SER9^ phosphorylation (Figure 2G). We confirmed these observations in two other *MYCN*-amplified neuroblastoma cell lines, IMR5 and SKN-Be2C, which showed similar patterns in sensitivity and biomarker modulation with all three drug treatments (Figure 2H). Furthermore, we treated the Kelly cell line at 2, 5 and 10μM with ZSTK474, which at higher concentrations can be a weak inhibitor of mTOR [59], and observed destabilization of MYCN, but only where mTOR activity was modulated (as measured through inhibition of both 4EBP1^THR37/46^ and RPS6^SER240/244^ (Figure 3A). Additionally, following cycloheximide treatment, the half-life of MYCN was shortened by NVP-BEZ235 and Torin1 but not by ZSTK474 (Figure 3B). Taken together, these data imply that the ability of PI3K/mTOR pathway inhibitors to eliminate MYCN protein correlates with effective targeting of both mTORC1 and mTORC2 activity.

### Blockade of mTOR stimulates proteasomal degradation of MYCN protein

We next assessed whether the ability of mTOR-kinase inhibitors to eliminate MYCN exhibited evidence of dependence on the ubiquitin-ligase, proteasome degradation pathway. Treatment with the proteasome inhibitor MG132 resulted in accumulation of phosphorylated MYCN and reversed the ability of NVP-BEZ235 to degrade MYCN protein (Figure 3C). NVP-BEZ235 efficiently and rapidly eliminated exogenously expressed MYCN-WT protein in SHEP WT cells (Figure 3D), an effect that was completely blocked by mutation of the CPD-domain T58 and S62 phosphorylation sites, despite evidence of excellent mTOR/PI3K pathway inhibition (Figure 3E). This suggests that the ability of PI3K/mTOR pathway inhibitors to eliminate MYCN is mediated by phosphorylation of the N-terminal FBW7 target CPD domain, since mutation of phosphorylation sites within the CPD completely reverses the ability of these drugs to degrade MYCN (compare Figure 3D, 3E). We therefore concluded that the elimination of MYCN protein caused by mTOR/PI3K pathway blockade is at least in part mediated through phosphorylation of MYCN T58/S62 phospho-residues.

### PI3K activity is not required for maintenance of cellular MYCN levels

**Figure 4:**
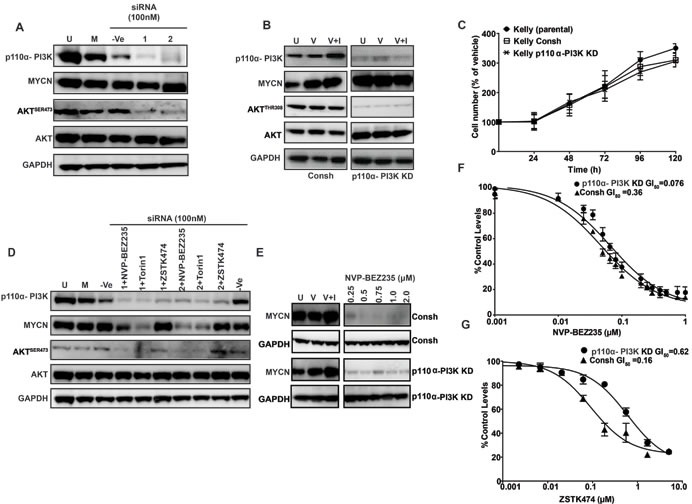
Role of the p110α-PI3K complex in MYCN stabilization Knockdown of p110 alpha PI3K with siRNA does not modulate MYCN levels in Kelly *MYCN*-amplified cells. **A.** Kelly neuroblastoma cells were transiently transfected with 100nM non-targeting (−Ve control) siRNA, or with 100nM of 2 single siRNA targeting P110α- PI3K. Cells were harvested 96 h post siRNA treatment and P110α- PI3K, AKT^SER473^ and MYCN protein levels were assessed *via* western blotting. **B.** Kelly neuroblastoma cells were stably transduced with control shRNA viral particles (Consh, left panel) or viral particles containing P110α- PI3K coding shRNA (Right panel) and PI3K-P110α, AKT^SER473^ and MYCN protein levels assessed *via* western blotting. **C.** Kelly neuroblastoma cells were stably transduced with control shRNA viral particles or viral particles containing P110α- PI3K coding shRNA and cell viability determined by SRB Kelly and Kelly CONSH shRNA neuroblastoma cell lines were used as controls. Values represent the averages of three independent assays. Error bars; standard deviation. **D.** Knockdown of p110α-PI3K does not affect MYCN destabilization induced by NVP-BEZ235 and Torin1. Kelly neuroblastoma cells were transiently transfected with P110 alpha PI3K targeting siRNA and at 3 h pre-harvest treated with NVP-BEZ235 (500nM), Torin1(500nM) or ZSTK474 (2μM). Cells were harvested 96 h post siRNA treatment and P110α- PI3K, AKT^SER473^ and MYCN protein levels were assessed *via* western blotting. **E.** Western blot analysis of Kelly Consh and Kelly PI3K-P110α shRNA cell lines treated with NVP-BEZ235 for 3 h. **F.**, **G.** GI_50_s as determined by trypan blue exclusion method in Kelly Consh and Kelly PI3K-P110α shRNA cell lines treated for 72 h with NVP-BEZ235 or ZSTK474. Mean GI_50_ and standard error from three independent assays are shown.

Our results indicate that PI3K inhibitors with weak activity against mTOR fail to target MYCN protein, implying that activity of the PI3K complex may not play a major role in stabilization of MYCN protein in neuroblastoma cells. To mechanistically address this possibility, we silenced p110α-PI3K expression in short- and long-term assays using siRNA and shRNA, respectively. Elimination of p110α activity blocked phosphorylation of AKT but failed to reduce cellular MYCN protein levels (Figure 4A, 4B) or alter cell proliferation (Figure 4C). In addition, the ability of NVP-BEZ235 to stimulate MYCN degradation in these cells (immunoblot Figure 4D, 4E) or their sensitivity to treatment with NVP-BEZ235 (GI_50_, as measured by 72 h SRB cytotoxicity assay, Figure 4F) was not altered by p110α knockdown, whereas sensitivity to treatment with ZSTK474 was significantly (6-fold) reduced (Figure 4G). This implies that the ability of combined PI3K/mTOR inhibitors to degrade MYCN protein is not dependent on intact p110β function, and furthermore that maintenance of MYCN protein levels in these cells largely proceeds through mTOR-dependent mechanisms.

### Elimination of MYCN protein requires concurrent inhibition of mTORC1 and mTORC2

**Figure 5:**
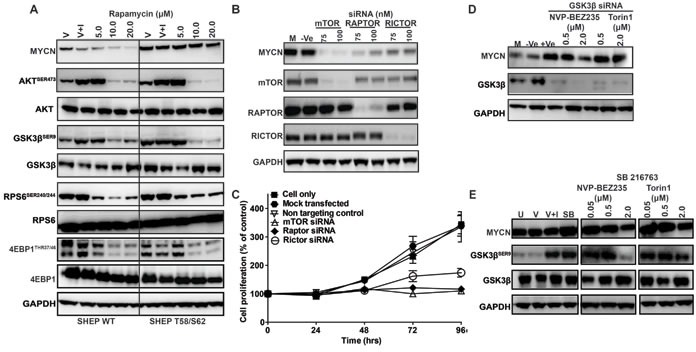
Role of mTOR and GSK3β in MYCN stabilization Impact of mTOR, RAPTOR and RICTOR knockdown on Kelly neuroblastoma cell growth and MYCN degradation. **A.** Western blot analysis of AKT^SER473^, GSK3β^SER9^, 4EPB1^THR37/46^, RPS6^SER240/244^ and MYCN levels after rapamycin treatment. SHEP WT and SHEP T58/S62 neuroblastoma cells were treated for 3 h with DMSO (V), with DMSO and IGF1 (V+I) or with rapamycin. Total AKT, GSK3β, S6 and 4EPB1 antibodies were used as controls for each corresponding phospho-specific antibody and GAPDH was used as a loading control. **B.** Kelly neuroblastoma cells were transiently transfected with 75nM and 100nM of mTOR, RAPTOR, RICTOR or non-targeting (Ve) siRNA and mTOR, RAPTOR, RICTOR and MYCN levels examined by western blot. **C.** Cell viability was determined by SRB assay. **D.** GSK3β inhibition stabilizes MYCN. Kelly neuroblastoma cells were transiently transfected with either a non-targeting (−Ve) or GSK3β targeting siRNA and at 3 h pre-harvest treated with NVP-BEZ235 (500nM) or Torin1 (500nM). Cells were harvested 96 h post siRNA treatment and GSK3β and MYCN protein levels assessed by western blot. **E.** Chemical inhibition of GSK3β in Kelly cells. Kelly cells were untreated (U), treated with DMSO (V), with DMSO and IGF1 (V+I), with GSK3β inhibitor SB216763 (SB) or with the indicated concentration of NVP-BEZ235 or Torin1 in the presence of SB216763. GSK3β^SER9,^ total GSK3β and MYCN protein levels were assessed by western blot. GAPDH was used as a loading control. See also Figure S2.

Although NVP-BEZ235 and Torin1, both ATP-competitive and potent inhibitors of PI3K/mTOR and mTORC1/mTORC2 respectively, most effectively targeted MYCN in our studies, incomplete (rapalogue) inhibitors of mTOR have been more commonly used in the clinical setting. To establish if incomplete inhibition of mTORC1 alone could be effective in destabilizing MYCN we used rapamycin at a low concentration, known to block mTORC1 but not mTORC2 [60]. We were only able to observe MYCN degradation using rapamycin at 10-20μM, a concentration at which total blockade of both mTORC1 (as measured by p4EBP1^THR37/46^) and mTORC2 (as measured by AKT^SER473^) occurs (Figure 5A). Similarly, the PI3K inhibitor ZSTK474 was only active against MYCN at high concentrations coinciding with inhibition of both mTORC1 (as measured by p4EBP1^THR37/46^ inhibition) and mTORC2 (as measured by AKT^SER473^ inhibition, Figure 3A). Furthermore, transient suppression of mTOR using siRNAs to target mTORC1 (RAPTOR) or mTORC2 (RICTOR) in Kelly cells partially reduced MYCN levels and targeting of mTOR kinase completely eliminated MYCN (Figure 5B, S2A-C), leading to rapid cell-killing (Figure 5C). Taken together these data imply that mTOR kinase plays a prominent role in the maintenance of MYCN stability and that complete blockade of both mTORC1 and mTORC2 is required to efficiently stimulate degradation of MYCN.

### The mTOR complex modulates GSK3β activity and regulates MYCN protein degradation

The beta isoform of glycogen synthase kinase (GSK3β) is a PI3K/mTOR pathway component and direct target of AKT that alters phosphorylation of MYC oncoproteins at the CPD. Phosphorylation of the CPD mediates complex formation between E3 ligases and MYC proteins, driving their degradation within the proteasome [61]. Activity of the mTORC2 (RICTOR/mTOR) complex specifically stimulates AKT^SER473^ phosphorylation [62]. High levels of mTORC2 activity lead to inactivation of GSK3β (preventing phosphorylation of MYCN and proteasomal degradation). In our hands, the ability of PI3K/mTOR pathway inhibitors (including NVP-BEZ235 and Torin1) to efficiently target MYCN required intact GSK3β activity (Figures 2 and 3). To test this observation, we knocked-out GSK3β with siRNA. Suppression of GSK3β increased stable intracellular levels of MYCN (Figure S2D, E) and both genetic (Figure 5D) and pharmacologic (Figure 5E) inhibition of GSK3β abrogated the ability of NVP-BEZ235, Torin1 (and other PI3K or mTOR inhibitors, data not shown) to degrade MYCN. These data establish that the mTOR complex, and mTORC2 in particular, plays a critical role in maintenance of cellular MYCN levels, requiring intact function of GSK3β, the activity of which appears to be rate-limiting for the ability of PI3K/mTOR inhibitors to stimulate MYCN degradation.

### *In vivo* inhibition of mTOR-kinase targets MYCN-driven tumors in animal models

**Figure 6:**
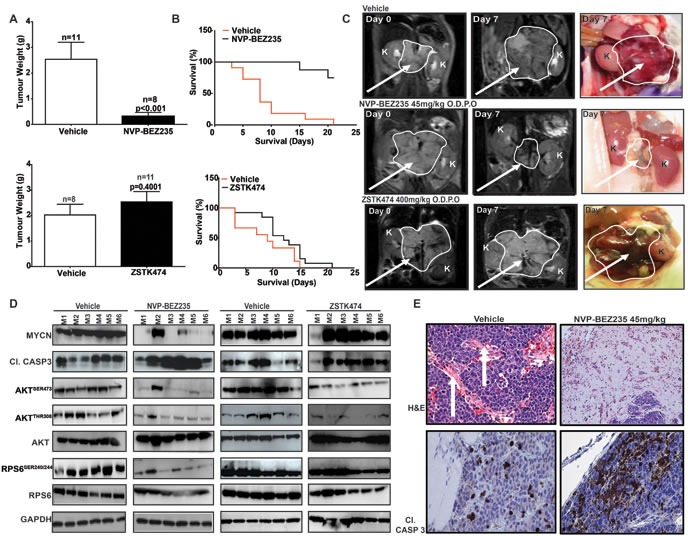
PI3K/mTOR pathway inhibition restricts the growth of MYCN-driven transgenic tumors Tumor-bearing animals transgenic for TH-*MYCN* were treated daily using oral gavage with 45mg/kg NVP-BEZ235 (*n* = 8), NVP-BEZ235 vehicle (*n* = 11), 400mg/kg ZSTK474 (*n* = 11) or ZSTK474 vehicle (*n* = 8). **A.** Average weight (g) of resected tumors relative to vehicle for NVP-BEZ235 (top panel) and ZSTK474 (lower panel). **B.** Kaplan-Meier 21 day survival curves for NVP-BEZ235 (top panel) or ZSTK474 (bottom panel). **C.** Representative T_2_-weighted MRI images and gross pathology of vehicle NVP-BEZ235 or ZSTK474 treated tumors at indicated times (arrow = tumor, K, kidney). **D.** Western blot analysis of tumors treated with NVP-BEZ235, ZSTK474 or respective vehicles. M1-M6 indicates tumor samples from cohorts of 6 individual animals, treated with either NVP-BEZ235, ZSTK474 or their respective vehicle controls. **E.** Tumor pathology (Hematoxylin & Eosin) and immunochemical staining (cl. Casp. 3) for vehicle (left panels) and NVP-BEZ235 treated (right panels) tumors. (White arrows = vascularization). See also Figure S3.

A stringent test of whether MYCN destabilization is a viable therapeutic strategy for MYCN-driven cancers is to establish whether murine tumors genetically-engineered to overexpress MYCN, together with primary human neuroblastoma tissue amplified for *MYCN*, are targeted *in vivo*. We assessed whether ZSTK474 and NVP-BEZ235 could inhibit tumor progression using a transgenic model of neuroblastoma (TH-*MYCN*), in which tumor formation is driven by tissue-specific overexpression of MYCN within murine neural crest [63]. Animals were treated for 21 days with 45mg/kg/day NVP-BEZ235 or 400mg/kg ZSTK474 [59, 64]. Both treatments were well tolerated (Figure S3A-C). Intra-tumoral levels of NVP-BEZ235 and ZSTK474 were verified by mass spectroscopy and the drugs were present in tumor tissue above concentrations required for inhibition of their respective targets (Figure S3D). Tumor mass response following necropsy (Figure 6A) and extension of survival (Figure 6B) were assessed as trial endpoints, and tumor regression was monitored *in vivo* using serial magnetic resonance imaging (MRI) (Figure 6C). Importantly, NVP-BEZ235 induced complete responses (by RECIST criteria) in the majority of treated tumors, with volume and mass reduction >75% (Figure 6A) translating to extension of survival (Figure 6B). Conversely, ZSTK474 lacked any anti-tumor activity (compare Figure 6A, 6B upper and lower panels) despite achieving intra-tumoral concentrations sufficient for inhibition of AKT phosphorylation (Figure 6D). In responding tumors, NVP-BEZ235 blocked both PI3K (AKT^SER473/308^) and mTOR signaling (RPS6^SER240/244^), resulting in almost complete elimination of MYCN protein (Figure 6D), and induction of apoptosis (cleavage of caspase 3) on immunoblot and by immunohistochemistry (Figure 6D, 6E). Interestingly, two mice in the NVP-BEZ235 treatment cohort experienced incomplete responses with partial tumor regression and progression, respectively (T2 and T4, Figure S3A, B), and in each case inhibition of PI3K (AKT^SER473/308^) and mTOR (RPS6^SER240/244^) was incomplete, with only partial reduction in MYCN levels (M2 and M4, Figure 6D). Consistent with the idea that complete blockade of mTOR is a requirement for therapeutic efficacy of PI3K/mTOR pathway inhibitors in the setting of MYCN-dependency, ZSTK474 exhibited little activity against mTOR signaling (RPS6^SER240/244^) and demonstrated no therapeutic benefit in this MYCN-driven model (Figure 6D).

To further confirm the efficacy of NVP-BEZ235 as a therapeutic for *MYCN*-amplified neuroblastoma in patients, we performed an *in vivo* preclinical trial using a primary xenograft model in which tumor tissue from a patient (SFNB-08) with high-grade, *MYCN*-amplified neuroblastoma was implanted orthotopically into the kidney capsule. Animals were treated with NVP-BEZ235 or vehicle for 14 days at 45mg/kg. Measurement of both tumor volume and weight again showed a significant decrease in tumor burden in NVP-BEZ235-treated animals in comparison to vehicle (Figure S4A, B). Immunoblotting of tumor tissue confirmed that NVP-BEZ235 blocked PI3K (AKT^SER473/308^, GSK3β^SER9^) and mTOR signaling (RPS6^SER240/244^, 4EBP1^THR37/46^) coincident with down-regulation of intra-tumoral MYCN protein (Figure S4C). In addition, Ki67 staining showed a significant reduction in cellular proliferation (Figure S4D). Taken together, these data illustrate the potency of the PI3K/mTOR inhibitor NVP-BEZ235 against two different MYCN-driven tumor models, establish that the mechanism of action relates to elimination of MYCN protein, and highlight the potential for treatment of MYCN-driven malignancies in children using clinically available inhibitors of mTOR.

## DISCUSSION

Oncogenic expression of MYC proteins is common in adult cancers and also occurs in neuroblastoma [1, 2], medulloblastoma [8], rhabdomyosarcoma [7] and retinoblastoma [9], four common pediatric solid tumors that together account for a major fraction of death from relapsed cancer in children. In these conditions, aberrant expression of MYCN is associated with high-risk disease, poor clinical prognosis and death due to metastatic relapse (reviewed in [4]). The prominent role of MYC proteins in oncogenesis, and the fact that MYCN expression is restricted to tumor tissue in several pediatric malignancies with poor outcome, implies that MYCN is likely to be an important therapeutic target, presenting a unique opportunity for targeted molecular therapeutic intervention in pediatric cancer [4].

That particular cancers can be addicted to oncogenic signaling changes induced by aberrant MYC expression is well-established through the use of oncogene-regulated mouse models [63, 65–75]. In this setting, repetitive genetic inactivation of MYC causes regression of several malignancies without evidence of acquired genetic resistance, and with reversible toxicity to normal organs [5, 76–79]. Collectively, these data underpin the rationale for direct pharmacologic inhibition of MYC, however in practice this has been difficult. Given the prominent role of MYCN in the genesis of aggressive pediatric tumors, we sought to identify existing therapeutics that could be useful to target MYCN indirectly, using neuroblastoma as a paradigm of a pediatric tumor that exhibits both clinical and experimental evidence of dependence and addiction to MYCN overexpression.

**Figure 7:**
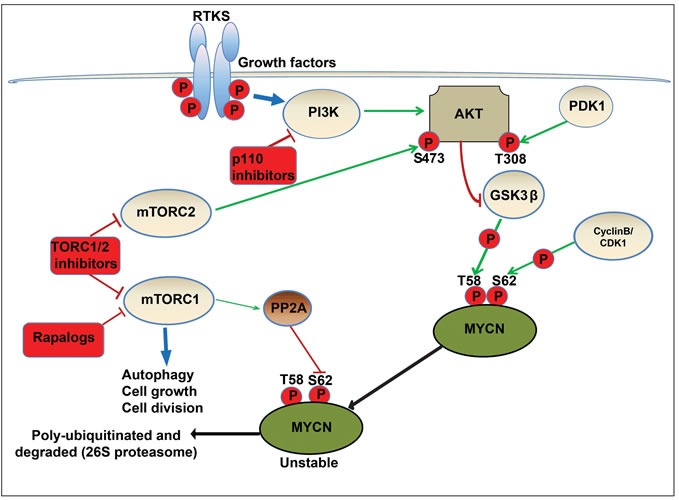
Schematic model of regulatory pathways involved in MYCN turnover In neuronal precursor cells, MYCN is initially phosphorylated at serine 62 (S62) by cyclin-dependent kinase 1/CyclinB. This acts as a priming phosphorylation that permits binding of GSK3β, PIN1 and PP2A. Subsequently, active GSK3β phosphorylates MYCN at threonine 58 (T58), yielding doubly phosphorylated MYCN. In a MYCN driven cancer cell model, RTKs activate PI3K, and the downstream target AKT, localising it to the membrane and allowing phosphorylation and activation of AKT at threonine 308 (T308) by PDK1. Active AKT phosphorylates and inactivates GSK3β, blocking phosphorylation of MYCN therefore preventing proteasomal degradation. The mTORC1 complex (mTOR-raptor) also directly phosphorylates and inhibits PP2A, enabling the accumulation of S62 phosphorylated and active MYCN. In addition, the mTORC2 complex (mTOR-RICTOR) phosphorylates and further activates AKT at serine 473 (S473). NVP-BEZ235 destabilizes MYCN by blocking activation of AKT (*via* inhibition of TORC1 and TORC2 complexes) and reactivating GSK3β, permitting phosphorylation of MYCN (T58) and initiating proteasomal degradation.

MYC-family proteins are oncogenically stabilized by altered phosphorylation within an n-terminal conserved phosphodegron domain (CPD). Binding to the CPD is a function of the ubiquitin ligases (FBW7, HUWE1) and is required for initiation of proteasomal degradation. Phosphorylation of conserved T58 and S62 residues within the CPD is regulated by CDK1 (a MAPK target), and by the PI3K/AKT-regulated targets GSK3β and mTOR, respectively (Figure 7). Based on knowledge of this mechanism, individual compounds which modulate GSK3β and target MYCN have been reported [13, 37, 80]. However, a comprehensive understanding of how PI3K/mTOR inhibitors target MYC proteins and a rationale for the selection of compounds likely to be clinically active against MYC-driven cancers is lacking. Given that pan-PI3K, dual PI3K/mTOR and TORC1/2 kinase inhibitors are progressing through early pediatric clinical evaluation, it is important to clearly define the patient population most likely to benefit from treatment and to prioritize inhibitors for clinical trials [52, 81]. To date, none of these compounds have been ranked for their potential to inhibit MYC/MYCN in pediatric cancers, although several drugs (rapamycin and the later generation rapalogue inhibitors temsirolimus and ridaforolimus) have been evaluated in the setting of relapsed solid tumors without a hypothesis relating to MYC or MYCN expression.

A practical cell-based approach to rank compounds with the ability to destabilize MYC protein would provide a valuable roadmap to prioritize drugs for early phase trials. We therefore designed a chemical-genetic screen using isogenic (SHEP neuroblastoma) cells varying in expression of wild-type or CPD-mutated, stabilized MYCN, and we assessed selective sensitivity to treatment with a library of 228 drugs or chemical probes [82, 83]. No statistically significant difference in proliferation rate was observed between SHEP cells expressing wild-type or stabilized MYCN in this screen, excluding the possibility that selective sensitivity related to changes in basal proliferation rate. However, we cannot discount the possibility that additional mechanisms associated with MYCN overexpression, and unrelated to stabilization of MYCN protein, could account for drug sensitivity in this screen. For example, MYC protein overexpression has been linked to defects in apoptosis [84], altered DNA damage repair responses [85], changes in chromosomal stability [86] and cell metabolism [87]. Many of these factors are the likely basis for previously described synthetic lethal relationships associated with MYCN expression. Nevertheless, we were able to identify several classes of small molecule inhibitor that selectively target cells expressing MYCN protein, and as proof-of-principle we characterized one class of compound that demonstrated considerable efficacy in two independent pre-clinical models of MYCN-driven neuroblastoma. We illustrate the feasibility of this mechanistic and translational approach in identifying regulators of MYCN stability, focusing on PI3K/mTOR and mTOR inhibitors as an effective class and mitigating the risk of off-target effects by using multiple inhibitors across the pathway, as well as siRNA silencing of pathway components. We focus on NVP-BEZ235 as an example of a selective PI3K/mTOR inhibitor that is able to suppress MYCN protein and induce growth-inhibitory effects in MYCN expressing cells, although several candidate inhibitors are now in clinical evaluation. From our data, the most effective inhibitors induce total blockade of both mTORC1 and mTORC2, which appear to regulate downstream processes that are key in controlling MYCN protein levels and cellular dependency on MYCN expression.

The basis for the relationship between MYCN expression and sensitivity to mTOR inhibition is unclear, but several possibilities are likely. In order to tolerate oncogenic overexpression of MYC, cancer cells must undergo a major remodeling of metabolic and translational pathways- both of which are regulated by mTOR [87]. Indeed, MYC has previously been shown to be involved in a feed-forward loop involving the translation machinery component eIF4F that links transcription and translation, regulating cell growth and protein synthesis [88, 89]. Uncoupling of this feed-forward loop *via* perturbations in expression of key proteins including those under the control of mTOR (such as 4EBP1) is a likely mechanism that fuels cancer cell growth and cellular addiction to MYC expression [16, 89–91]. Finally, neuroblastomas with high levels of MYCN expression exhibit defined metabolic deficiencies in glucose metabolism (Warburg physiology) and are polyamine-dependent under hypoxic conditions [92, 93]. MYC directly interacts with HIF-1 linking altered cellular metabolism to tumorigenesis by regulating genes involved in the biogenesis of ribosomes, mitochondria, and regulation of glucose and glutamine metabolism [94, 95].

Advances in medicinal chemistry have delivered potent small-molecules to clinical use that have the ability to achieve effective *in vivo* blockade of major oncogenic signaling pathways through either direct targeting of oncoproteins or through synthetic lethal relationships exposed by oncogene expression [83]. The availability of multiple clinical inhibitors that target pathways of high relevance to cancer treatment mandates thorough mechanistic prioritization. Here we show that within the class of available inhibitors of PI3K/mTOR pathway, compounds that selectively block mTORC1/mTORC2, such as the combined PI3K/mTOR inhibitor NVP-BEZ235, efficiently target MYCN protein stability and cause *in vivo* regression of neuroblastoma, a MYCN-driven pediatric tumor. Selective inhibitors of PI3K do not exhibit this activity. This has important implications for planned trials of these compounds in pediatric cancer, since multiple PI3K, mTOR and dually targeted PI3K/mTOR drugs are already in active clinical evaluation. The success of pediatric trials utilizing these agents will depend as much on careful selection of the appropriate agent as on the target patient population likely to benefit from treatment.

## MATERIALS AND METHODS

### Cell lines and reagents

Kelly, SH-SY5Y, SHEP, SK-N-SH, IMR32, SKN-Be2C, IMR5 and SK-N-AS human neuroblastoma cell lines were obtained from the University of California at San Francisco Cell Culture Facility (San Francisco, CA) and from the American Type Culture Collection (Manassas, VA). Cells were grown in DMEM or RPMI containing 10% fetal bovine serum (PAA “Gold”). In specified experiments, cells were serum starved in 0.2% FCS for 6 h before analysis and treated with recombinant human insulin-like growth factor-1 (IGF-1; Sigma) at 50ng/mL for 30 min before harvesting. NVP-BEZ235 (Novartis), Torin1 (Nathaniel Gray), staurosporine (Alexis Biochemicals), ZSTK474 (Alexis Biochemicals), GSK3β inhibitor (Calbiochem), TGX221 (Selleck chemicals), PIK90 (Selleck chemicals) and Rapamycin (Selleck chemicals) were all prepared as a 10mM stock solution in 100% DMSO. Working solutions were prepared freshly by dilution in 100% DMSO prior addition to the cell media at a final concentration of 0.1% DMSO. SHEP cells were stably transfected with constructs wild-type or mutant for MYCN and appropriate clones were screened and selected essentially as described previously [37]. Cells were regularly screened for Mycoplasma using a PCR-based assay (VenorGem, Minerva Biolabs).

### *In vitro* proliferation and inhibitor treatment

Cells were seeded in triplicate wells of 96-well flat bottom culture plates (3-6 × 10^3^ per well) and allowed to attach for 24 h to ensure exponential growth at the time of treatment. Cells were then incubated for 72 h in the presence of increasing concentrations of indicated inhibitors. Cell viability and median-effect concentration affecting growth (GI_50_) was determined using the SRB (Sulforhodamine B colorimetric assay) and GI_50_ values were derived as described previously [96]. Apoptosis was assessed by caspase cleavage. The activities of caspase-3 and −7 were measured by luminescence detection using the Caspase-Glo® 3/7 assay kit (Promega, Inc.). The assay provides a luminogenic caspase-3/7 substrate in a reagent that when added to cells results in cell lysis, followed by caspase cleavage of the substrate and generation of a luminescent signal produced by luciferase. The assay was performed according to the manufacturer's instructions. The mean difference was calculated using Student's t-test. Values were standardized to the maximal effect of Staurosporine (2μM).

### Kinase inhibitor screen

SHEP WT and SHEP T58/S62 were seeded in 384-well plates for 48 hours before treatment with 40, 200 and 1000nM of 228 kinase inhibitors and chemical probes for 96 hours [82, 97–99]. Cell viability was determined using CellTiter-blue reagent (Promega, Inc.). The Z-factor was >0.5 for all plates analyzed. The data was plotted as a ratio of SHEP T58/S62:SHEP WT.

### Immunoblotting

Inhibitor-treated cells were lysed and suspended in non-denaturing lysis buffer (Cell Signaling Technology, Danvers, MA) containing 1x concentrate protease and phosphatase inhibitor cocktail tablets (Roche, Lewes, UK). Samples were transferred to polyvinylidene difluoride membranes, which were incubated in a blocking buffer (5% dried milk in TBS or 3% ECL advance blocking reagent) and probed with primary antibody in blocking buffer overnight at 4°C. Proteins were detected with HRP conjugated secondary antibody (DAKO) and visualized with enhanced chemiluminescence reagents (GE Healthcare). Antibodies used were as follows: Rabbit anti MYCN polyclonal antibody (Santa Cruz Biotechnology, Santa Cruz, CA), mouse anti MYCN monoclonal antibody (Calbiochem, Merck KGaA, Darmstadt, Germany), rabbit anti-phospho MYCN (S54) polyclonal antibody (Bethyl Labs, Montgomery, TX), rabbit anti-phospho c-Myc (T58/S62) polyclonal antibody (Cell Signaling Technologies, Danvers, MA), Rabbit and mouse antibodies specific for total and phosphorylated forms from CST: AKT (#2965, #9267, #4058, #9272, #9271, #4056), ribosomal protein S6 (#2317, #2211), P70S6K (#9204, #2708), 4EBP1 (#9644, #9459), GSK3β (#9323, #9315), cleaved caspase-3 (#9664), Raptor (#2280), Rictor (#2114), mTOR (#2983) and GAPDH (#2118). Phosphorylation of AKT^SER473^, GSK^SER9^, RPS6^SER240/244^ and P70S6K^SER421/424^ was also determined using an electrochemiluminescent immunoassay (MesoScale Discovery) as described previously [44].

### Transfection of Kelly neuroblastoma cells with siRNA

MYCN, GSK3β, mTOR, RAPTOR or RICTOR single or SMARTpool siRNA (Dharmacon) and PI3K-P110α single siRNA (Ambion) was introduced into the cells by complex formation with Lipofectamine 2000 lipid transfection reagent (Invitrogen) according to transfection recommendations of the manufacturer. Lentiviral packaging vectors pMD2.G (3μg), pMDLg/pRRE (5μg), pRSV-REV (2.5μg) (Addgene) and the MYCN shRNA vector (10μg, TRCN0000020696, Sigma-Aldrich, UK) or control shRNA vector (10μg, SHC002 Sigma-Aldrich), also in complex with lipofectamine, were transfected into the HEK293T packaging cell line seeded in T75cm^2^ tissue culture flasks. Twenty-four hours after transfection, the culture media were replaced by fresh media corresponding to the cell line being transduced. After another 24 h of incubation, the lentiviral particle containing media were clarified by centrifugation and passed through a Millex HV 0.45 μm PVDF filter (Millipore, Bedford, MA, USA). The viral titre was calculated using the HIV p24 ELISA (Cambridge Biosciences) and viral aliquots stored at −80°C. KELLY, SHEP WT and SHEP T58/S62 cells were transduced with lentivirus at a calculated multiplicity of infection (MOI) of 10 (assuming 50% packaging efficiency) in the presence of polybrene (4μg/ml). The following day the virus-containing media were replaced with normal growth media and 24 h later, knock down cells were selected using puromycin (5μg/ml continuous treatment for at least 1 week).

### *In vivo* transgenic experiments

Transgenic TH-*MYCN* animals with palpable tumors (40-80 days old) were randomized and treated with 45mg/kg NVP-BEZ235, 400mg/kg ZSTK474, or vehicle. NVP-BEZ235 and ZSTK474 were administered via oral gavage. To assess the pharmacokinetic and pharmacodynamic profiles of NVP-BEZ235, plasma and tumor samples were harvested at 1 hour and 6 hours following dosing. Mice were bled by cardiac puncture, and plasma samples were collected and frozen at −20°C until analysis. Tumors were dissected, divided into 3 approximately equal pieces, and snap frozen in liquid nitrogen until analysis. For pharmacodynamic studies, tumors were homogenized using T-PER buffer™ (Thermo Scientific), 1x concentrates protease and phosphatase inhibitor cocktail tablets (Roche, Lewes, UK). Protein content was measured using Bradford reagent and samples were analyzed using immunoblotting as described previously. MRI was performed on a 7T Bruker horizontal bore microimaging system (Bruker Instruments, Ettlingen, Germany) using a 3cm birdcage coil. Anatomical T_2_-weighted coronal images were acquired from twenty contiguous 1mm thick slices through the mouse abdomen, from which tumor volumes were determined using segmentation from regions of interest drawn on each tumor-containing slice At necropsy, tumors were excised, measured, weighed, and snap frozen. Significance analysis was performed using the Student's *t* test. Mice were monitored daily and tumor size was palpated and animal weight measured daily. Animals were treated in accordance with local and national animal welfare guidelines [100].

### *In vivo* orthotopic experiments

A human *MYCN*-amplified primary tumor designated SFNB-08 was obtained from a patient with high-grade neuroblastoma, previously treated with cyclophosphamide, topotecan, cisplatin, etoposide, doxorubicin, and vincristine. Tumor pieces (4mm^3^) were implanted into the kidney capsule of nude mice. After 7 days, mice were treated with either vehicle PEG300 control or NVP-BEZ235 (45mg/kg in PEG300) daily for 14 days. Tumors were collected on the last day of treatment, weight and volume (as measured by digital caliper) were recorded and tissues taken for histopathology and western blotting

### Drug measurements

Concentrations of NVP-BEZ235 in biological samples were determined using liquid chromatography/mass spectrometry (LC-MS). Drug was extracted using 3-4 vol methanol containing 500ng/ml Olomoucin used as internal standard. Chromatography carried out on a Kinetics C18 column (5.0 cm × 2.1 mm ID, 2.6-μm particle size; Phenomenex) using a 1290 LC system with a gradient mobile phase of 0.1% formic acid/methanol at 0.6 mL/min over 10 minutes. Detection was by LC-MS using a Agilent 6410triple quadropole in which the analyte was ionized by electrospray interface in positive mode (gas temperature 300 flow 12 l/min, nebulizer 40psi, capillary voltage 4000V. The following transition were monitored [M + H]^+^472.2 to 454.2 at 50V collision energy for NVP-BEZ235and [M + H]^+^ 299.1 to 177.1 at 30V collision energy for Olomoucin. The assay was linear in the range of 10-5000 nM.

### Statistical analyses

We analyzed quantitative results either by one-way analysis of variance (multiple groups) or t test (two groups). We carried out survival analysis using Kaplan-Meier log-rank test. We considered a difference with *P* < 0.05 (two-sided) statistically significant.

## SUPPLEMENTARY MATERIAL FIGURES AND TABLES


