# Supplementary material for: Inhibition of mTOR-kinase destabilizes MYCN and is a potential therapy for MYCN-dependent tumors
Source: Oncotarget. 2016 Jul 12;7(36):57525–44. doi: 10.18632/oncotarget.10544 (PMC5295370; doi:10.18632/oncotarget.10544)
Supplement: Supplementary file 1 [file oncotarget-07-57525-s001.pdf]

Inhibition of mTOR-kinase destabilizes MYCN and is a potential therapy for MYCN-dependent tumors

Supplementary Material

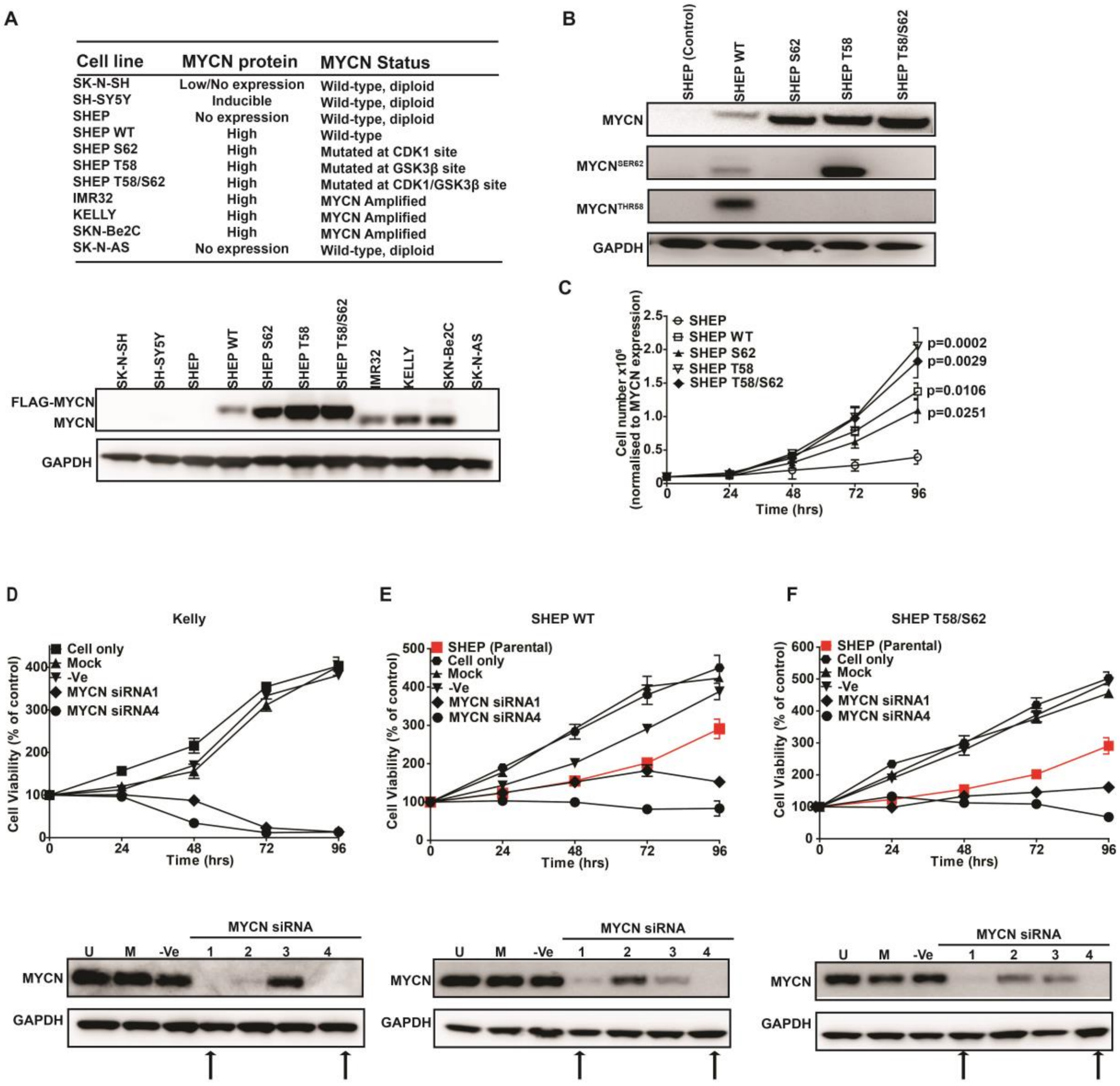

Figure S1: Identification of PI3K/mTOR inhibitors that selectively target MYCN.

A. Description and MYCN protein expression of a panel of neuroblastoma cell lines representing a spectrum of MYCN protein expression and/ or engineered to stably express murine MYCN constructs wild-type or mutated at critical phospho-residues. **B.** Western blotting analysis of

MYCN, MYCN^{SER62} MYCN^{THR58} protein levels in SHEP NB cells expressing murine MYCN wild-type or mutated at T58/S62 phospho-residues. **C.** Cell growth as determined by the Trypan blue exclusion method over a time course of 96 h in parental SHEP and SHEP MYCN phosphomutant cell lines. Depletion of MYCN selectively reduces cell viability of Kelly, SHEP WT and SHEP T58/S62 NB cells. **D-F.** Kelly SHEP-WT and SHEP T58/S62 NB cells were mock transfected with Lipofectamine 2000 (M), transfected with 100nM of non-targeting siRNA (-Ve), or with multiple MYCN targeting siRNA (100nM) and cell viability and MYCN expression levels determined by Trypan blue exclusion assay and immunoblotting respectively. Values represent the averages of three independent assays. Error bars; standard deviation.

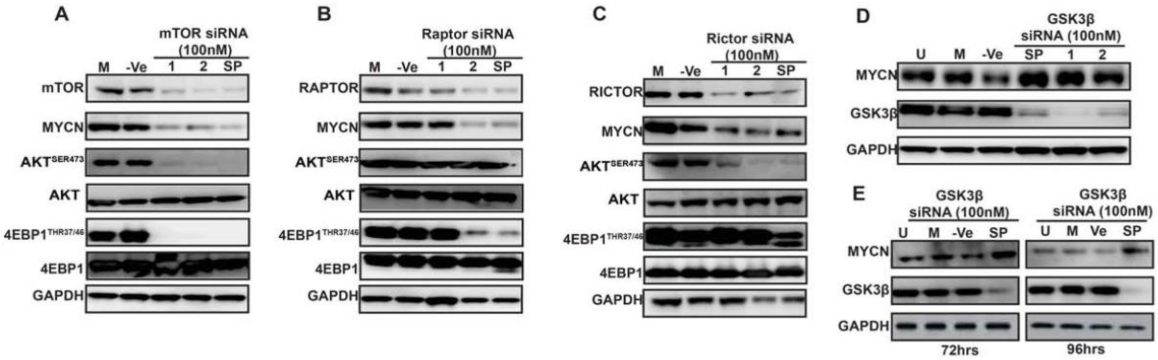

Figure S2: Effective targeting of MYCN stability requires mTOR inhibition.

Knockdown of mTOR RAPTOR and RICTOR destabilizes MYCN and modulates TORC1/2 targets in Kelly NB cells. **A.** Kelly NB cells were mock transfected with Lipofectamine 2000 (M), transfected with 100nM of non-targeting siRNA (-Ve), with 2 independent mTOR targeting siRNA (100nM) or with 4 pooled mTOR targeting siRNA (100nM) and protein levels of MYCN, mTOR, AKT^{SER473}, and 4EPB1$^{THR37/46}$ was assessed by western blot analysis. **B.** Kelly NB cells were mock transfected with Lipofectamine 2000 (M), transfected with 100nM of non-targeting siRNA (-Ve), with 2 independent RAPTOR targeting siRNA (100nM) or with 4 pooled

RAPTOR targeting siRNA (100nM) and protein levels of MYCN, RAPTOR, AKT^{SER473}, and $4EPB1^{THR37/46}$ was assessed by western blot analysis. **C.** Kelly NB cells were mock transfected with Lipofectamine 2000 (M), transfected with 100nM of non-targeting siRNA (-Ve), with 2 independent RICTOR targeting siRNA (100nM) or with 4 pooled RICTOR targeting siRNA (100nM) and protein levels of MYCN, RICTOR, AKT^{SER473}, and $4EPB1^{THR37/46}$ was assessed by western blot analysis. **D.** GSK3β modulation is rate-limiting for MYCN destabilization. Kelly NB cells were mock transfected with Lipofectamine 2000 (M), transfected with 100nM of non-targeting siRNA (-Ve), with 2 independent GSK3β targeting siRNA (100nM) or with 4 pooled GSK3β targeting siRNA (100nM) and protein levels of MYCN and GSK3β was assessed by western blot analysis. **E.** Western blot analysis of MYCN levels at 72 and 96 h post GSK3β depletion. Kelly cells were; untreated (U), mock transfected with Lipofectamine 2000 (M), transfected with 100nM of non-targeting siRNA (-Ve), or 100nM pooled GSK3β siRNA and protein levels assessed via western blotting.

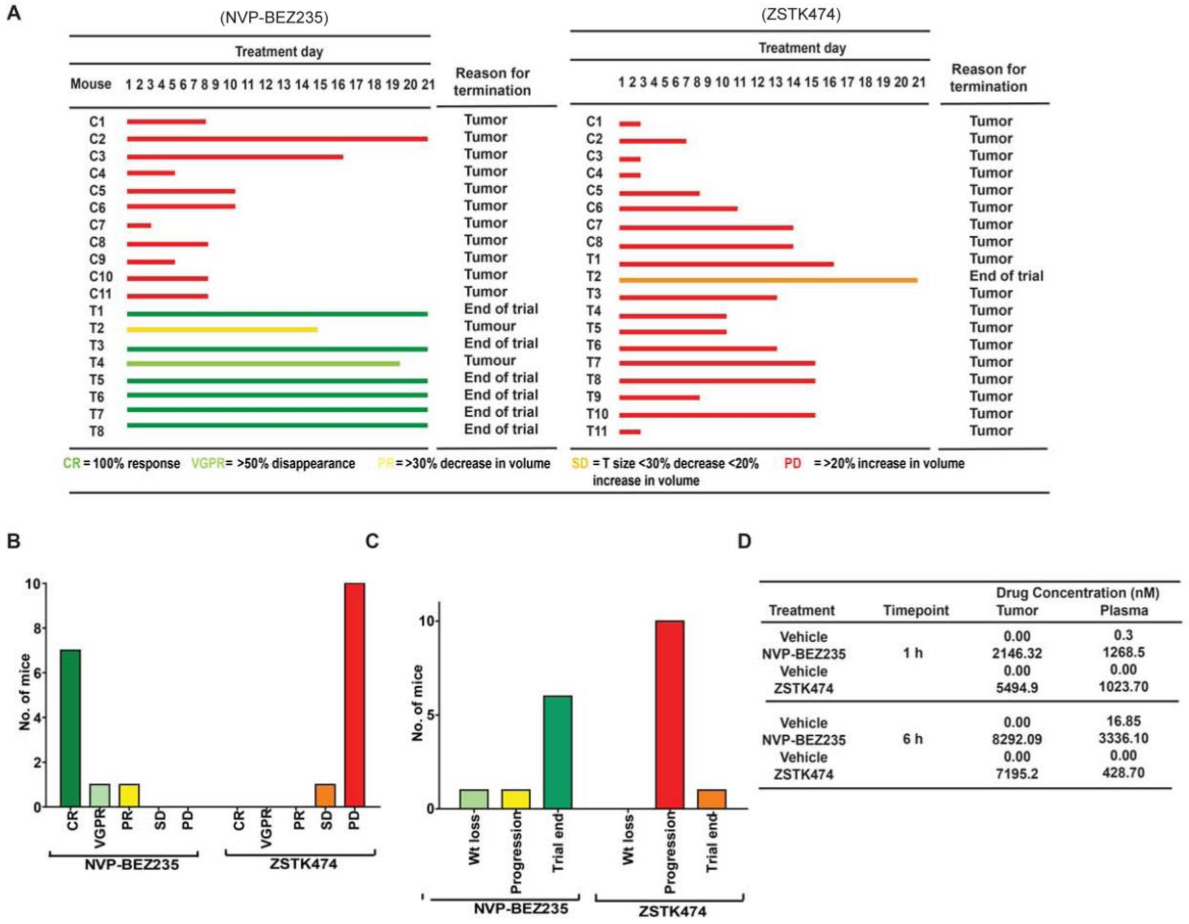

Treatment	Timepoint	Drug Concentration (nM) Tumor	Plasma
Vehicle		0.00	0.3
NVP-BEZ235	1 h	2146.32	1268.5
Vehicle		0.00	0.00
ZSTK474		5494.9	1023.70
Vehicle		0.00	16.85
NVP-BEZ235	6 h	8292.09	3336.10
Vehicle		0.00	0.00
ZSTK474		7195.2	428.70

Figure S3: PI3K/mTOR inhibition is effective against MYC-driven transgenic tumors.

Tumor-bearing animals transgenic for TH-*MYCN* were treated daily using oral gavage with 45mg/kg NVP-BEZ235 (n=8), NVP-BEZ235 vehicle (n=11), 400mg/kg ZSTK474 (n=11) or ZSTK474 vehicle (n=8). Treatment was for an average of 21 days, at which time the animals were killed. **A.** RECIST assessment of response of the individual animals from Fig 6 to NVP-BEZ235 (left panel) or ZSTK474 (right panel). **B.** Summary of RECIST response of animals. **C.** Summary of toxicity of NVP-BEZ235 or ZSTK474 in comparison to vehicle. **D.** Pharmacokinetic analysis of NVP-BEZ235 and ZSTK474 inhibitor levels in tumor tissue and plasma.

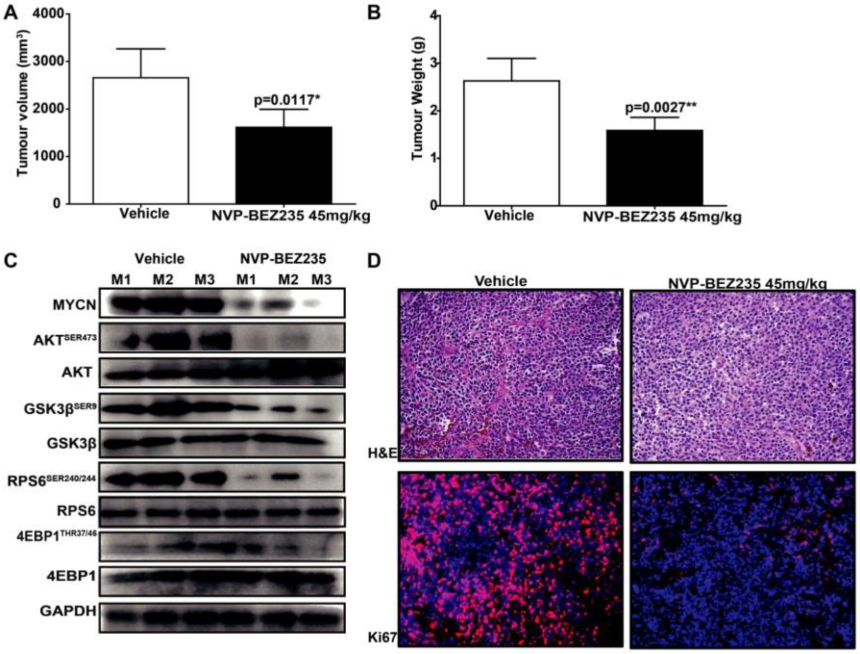

Figure S4: PI3K/mTOR inhibition is effective against MYC-driven orthotopic tumors.

A human *MYCN*-amplified primary tumor designated SFNB-08 was obtained from a patient with high-grade neuroblastoma that had been previously treated with cyclophosphamide, topotecan, cisplatin, etoposide, doxorubicin, and vincristine. Tumor pieces (4mm^3) were implanted into the kidney capsule of nude mice. After 7 days, mice were treated with either vehicle PEG300 control or NVP-BEZ235 (45mg/kg in PEG300) daily for 14 days. Tumors were collected on the last day of treatment, their weight and volume (as measured by digital caliper) recorded and tissues taken for histopathology and western blotting. **A.** Average volume (mm^3) of resected tumors from either NVP-BEZ235 or vehicle control treated animals **B.** Average weight (g) of resected tumors from either NVP-BEZ235 or vehicle control treated animals. **C.** Western blot analysis of tumors treated with NVP-BEZ235 or vehicle control. M1-M5 indicates tumor samples from cohorts of 5 individual animals, treated with either NVP-BEZ235 or vehicle control. **D.** Tumor pathology (Hematoxylin & Eosin) and immunochemical staining (cl. Casp. 3) for vehicle (left panels) and NVP-BEZ235 treated (right panels) tumors. (White arrows = vascularization).

Supplementary table legends

Table S1: Published IC_{50} for small molecule PI3K/mTOR inhibitors

				IC50 (nM)				
Inhibitor	PI3Kα	PI3Kβ	PI3Kγ	PI3Kδ	Torc1	Torc2	mTOR	DNA-PK
LY294002	500	580	12	600	-	-	-	-
ZSTK474	16	44	49	4.6	-	-	>10000	-
PIK90	11	350	18	58	-	-	l	13
PI-103	2	3	15	3	20	83	20	2
TGX221	5000	5	3500	100	-	-	-	-
Torin1	-	-	1800	-	10	10	10	-
Rapamycin	-	-	-	-	-	-	-	-
NVP-BEZ235	4	75	5	7	10	10	10	-

- Including PI3K-p110 isoforms and mTOR complex components (Jackson et al., 2005; Kong and Yamori, 2007;

Liu et al., 2010; Maira et al., 2008; Raynaud et al., 2009; Yaguchi et al., 2006)

Table S2: Treatment of NB cell lines with a panel of PI3K/mTOR pathway inhibitors

Cell line	MYCN Expression	MYCN Status	TGX221	PIK90	Rapamycin	ZSTK474	Torin1	NVP-BEZ235
**Kelly**	High	Wild-type	1.03+/-0.04	0.33+/-0 .03	8.83+/-0 .82	0.32+/0.03	0.04+/-0.01	0.05+/-0 .01
**IMR32**	High	Wild-type	0.23+/-0.02	0.41+/-0.02	12.39+/0 .18	0.27+/-0 .12	0.78+/0.01	0.062+/-0 .01
**SKN-Be2C**	High	Wild-type	0.78+/-0.22	0.18+/-0.07	13.13+/-0.15	0.39+/-0.04	0.19+/0.06	0.038+/-0 .03
**IMR5**	High	Wild-type	0.75+/-0.19	0.56+/0 .06	12.91+/-0 .15	0.29+/-0.10	0.15+/0.09	0.071+/-0.02
**SH-SY5Y**	Inducible	Wild-type	1.95+/-0.32	1.64+/-0 .19	10.31+/-0.67	0.79+/-0.06	0.42+/-0.51	0.49+/-0 .035
**SK-N-SH**	Low	Wild-type	1.87+/-0.11	0.97+/-0 .18	10.29+/-0 .81	0.98+/-0.14	1.34+/-0.05	0.42+/-0 .04
**SHEP-WT**	High	Wild-type	2.25+-0.21	0.92+/-0 .03	10.72+/-0 .62	0.51+/-0.041	0.2+/-0 .01	0.18+/-0.028
**SHEP-S62**	High	CDK1 mut	2.84+/-0.07	2.03+/-0 .07	11.01+/-0.23	3.87+/-0.54	1.89+/-0.31	1.18+/-0.31
**SHEP-T58**	High	GSK3 mut	1.97+/-0.07	1.93+/-0 .04	10.46+/-0.18	3.53+/-0.25	2.03+/-0.12	1.21+/-0 .14
**SHEP-T58/S62**	High	CDK1/GSK3 mut	2.63+/-0.05	4.93+/-0.21	12.93+/-0.72	6.03+/-0.87	2.36+/-0 07	1.88+/-0 .51
**SHEP**	No expression	No Mycn	4.91+/-0.14	3.92+/-0.31	11.23+/-0.54	2.87+/-0.48	1.23+/0.12	3.52+/-0 .27
**SK-N-AS**	No expression	No Mycn	0.23+/-0.24	0.79+/-0.11	14.91+/-0.28	1.23+/-0.35	2.21+/-0.38	0.41+/-0 .24

Cell viability 72h post treatment as determined by SRB assay for an isogenic panel of NB cell lines expressing varying levels of MYCN or phospho-stabilized MYCN proteins. Values are mean 72h GI_{50} derived from three independent assays. Errors are standard deviations. Cells engineered to express MYCN (SHEP-WT), phospho-mutated MYCN (SHEP-T58/S62) or native MYCN amplified cells with high MYCN protein levels are boxed.
